# Munchausen's syndrome presenting as rectal foreign body insertion: a case report

**DOI:** 10.1186/1757-1626-1-243

**Published:** 2008-10-16

**Authors:** Shakeeb A Khan, Christine A Davey, Shamsul A Khan, Peter J Trigwell, Srinivas Chintapatla

**Affiliations:** 1 Department of Surgery, York District Hospital, Wiggington Road, York, UK; 2 North Yorkshire Alliance Research and Development Unit, York District Hospital, Wiggington Road, York, UK; 3 Department of Liaison Psychiatry, Leeds General Infirmary, Great George Street, Leeds, LS1 3EX, UK

## Abstract

**Background:**

This case report shows that Munchausen's syndrome can present as rectal foreign body insertion. Although the presentation of rectal foreign bodies has frequently been described in the medical literature, the insertion of foreign bodies into the rectum for reasons other than sexual gratification has rarely been considered.

**Case presentation:**

A 30 year old, unmarried Caucasian male presented with a history of having been sexually assaulted five days earlier in a nearby city by a group of unknown males. He reported that during the assault a glass bottle was forcibly inserted into his rectum and the bottle neck broke. On examination, there was no evidence of external injury to the patient. Further assessment lead to a diagnosis of Munchausen's syndrome. The rationale for this is explained. A description and summary of current knowledge about the condition is also provided, including appropriate treatment approaches.

**Conclusion:**

This case report is important because assumptions regarding the motivation for insertion of foreign bodies into the rectum may lead to the diagnosis of Munchausen's syndrome being missed. This would result in the appropriate course of action, with regard to treatment, not being followed. It is suggested that clinicians consider the specific motivation for the behaviour in all cases of rectal foreign body insertion, including the possibility of factitious disorder such as Munchausen's syndrome, and avoid any assumption that it has been carried out for the purpose of sexual gratification. Early involvement of psychiatrists is recommended. Cases of Munchausen's syndrome presenting as rectal foreign body insertion may be identified and addressed more effectively using the approach described.

## Background

This case report highlights the previously unreported scenario of rectal foreign body insertion being a manifestation of Munchausen's syndrome. The literature is reviewed and the features that lead to this primary psychiatric disorder and its management on a medical ward are discussed.

Rectal foreign body insertion has frequently been described in the medical literature. Indeed, Haft and Benjamin refer to a case as long ago as the sixteenth century [1]. Sexual pleasure, either as autoeroticism or as part of a consensual sexual act, is the reason most often cited [2-7] although in a review of 186 case reports Busch and Starling note the difficulty of generalising about the sexual preferences of male patients presenting with rectal foreign bodies [8]. Other reported circumstances include assaults [3], accidents [9], smuggling [10], iatrogenic mishaps [11] and self-treatment e.g. to relieve constipation or other anal symptoms [12,13].

The insertion of foreign bodies in the rectum for psychological reasons other than sexual gratification has rarely been considered, though there have been some brief references in the literature [14,15]. Clarke et al suggest that 'attention-seeking' is the reason for foreign body insertion in three of the 13 patients in their case series [16]. Here we present a case in which Munchausen's syndrome is the most likely explanation for the rectal insertion of a foreign body. On a comprehensive literature review only one previous case report was found which mentioned Munchausen's as a possible cause of rectal foreign body insertion [17].

## Case presentation

A 30-year-old, unmarried Caucasian male presented with a history of having been sexually assaulted five days earlier in a nearby city by a group of unknown males. Reportedly they kicked and stamped on him and during the assault a glass bottle was forcibly inserted into his rectum and the bottle neck broke.

The patient appeared very agitated and requested that only females be involved in his care. The residents on call for surgery were both female. When the male on call surgical consultant arrived the patient became very agitated and uncooperative.

**Figure 1:**
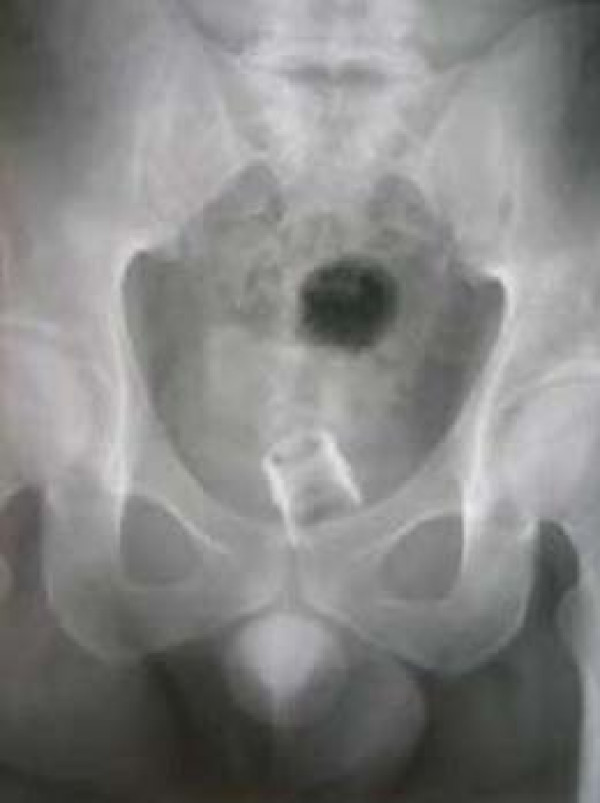
Plain X – Ray showing bottle fragment *in situ.*

On examination, there was no evidence of external injury to him. His abdomen was soft and showed a well-healed lower midline incision. He attributed this scar to a laparotomy done for abdominal pain in his childhood. Plain X-Ray of his abdomen showed the broken off upper part of a glass bottle (Figure 1)

**Figure 2:**
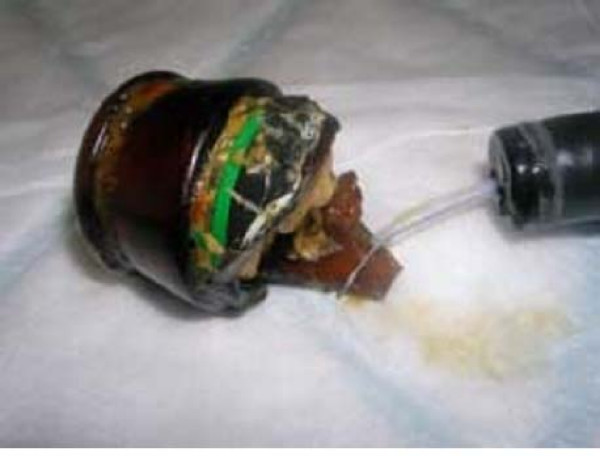
Endoscopic snare with bottle fragment.

**Figure 3:**
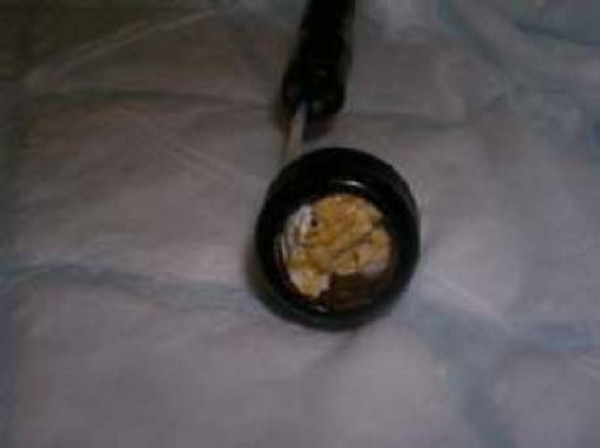
Bottle neck packed with paper.

The patient refused a rectal examination on the ward. On examination under anaesthesia, in the lithotomy position, no evidence of perianal trauma was found. A flexible endoscope was inserted and the broken glass bottle was confirmed to be in the sigmoid colon. An endoscopic snare was passed and the broken glass bottle retrieved (Figure 2). Repeat endoscopy did not show any damage to the mucosa. The neck of the glass bottle was found to have a roll of paper inserted within it (Figure 3).

The following morning the patient complained of pain in his abdomen. His abdomen was tender though there was no guarding or rigidity. Due to the possibility of rectal perforation, a CT scan of his abdomen and pelvis was arranged.

Due to the unusual circumstances, the case was discussed with a second surgical consultant within the same department. Surprisingly, he recognised the patient from a previous post at a nearby hospital. The same man had presented under a similar sounding name with an almost identical history. On that previous occasion he had also claimed to have been sexually and physically assault in a car park, during which time a bottle top was reportedly inserted into his rectum. This was removed under general anaesthesia shortly after which he self-discharged, with no follow-up being possible as he had no General Practitioner.

During the current admission, an attempt was made to telephone the person the patient had nominated as his next of kin. However, the number proved to be of someone in a foreign country who denied knowing the patient and informed us that he had received a similar enquiry, about a month ago from another hospital in the UK.

A psychiatric consultation was requested upon which the patient became angry and refused further treatment. However, with the encouragement of a nurse, he later agreed to see the psychiatrists. The psychiatry team did not find any evidence of a formal psychiatric disorder, other than the possibility of Munchausen's syndrome. When asked about the possible earlier presentation at another hospital the patient became very angry and took his own discharge against medical advice.

He had told us that he was not registered with a GP and later it was discovered that the home address he had given at registration was a fictitious one. During his stay he had no visitors and it was not possible to contact any true next of kin, as the names and addresses given were also false.

## Discussion

Our patient gave a history of sexual assault but several features of his presentation, which will be outlined below, led us to favour a primary psychiatric diagnosis of Munchausen's syndrome.

Munchausen's syndrome was first described by Asher in 1951 taking the name from Baron von Munchausen, an 18^th ^century gentleman renowned for wandering from city to city and telling dramatic stories about his life [18]. In psychiatric classification Munchausen's syndrome is a severe form of *factitious disorder*, where symptoms (physical or psychiatric) are produced deliberately without evidence of external incentives [19]. The main aims in Munchausen's syndrome are of obtaining the benefits of the 'sick role' and gaining pleasure from deceiving doctors [20]. Factitious disorders can be distinguished from other psychological causes of medically unexplained symptoms: somatisation and malingering. In somatisation, the physical symptoms are not produced deliberately and are an unconscious manifestation of psychological distress. In malingering, symptoms are produced deliberately for an identifiable external incentive or gain e.g. money, shelter, or court avoidance [19] and there is a wish to avoid any invasive procedures [21].

The prevalence of Munchausen's syndrome, and of factitious disorders generally, is difficult to ascertain and is likely to be underreported. In Sutherland's review of 1,200 referrals to a hospital liaison psychiatry department, only 0.8% of patients had factitious disorder with physical symptoms [22]. Medical and surgical teams may thus be unfamiliar with this condition and may not refer to the psychiatry team stating factitious disorder as the reason for the referral [21]. Patients may receive alternative non-specific medical or psychiatric diagnoses such as 'abdominal pain' or 'personality disorder' [21].

There are various approaches to the diagnosis of Munchausen's Syndrome. O'Flynn et al. describe the following eight principal features: "pathological lying with the presentation of the history in a dramatic, vague and inconsistent manner; evidence of prior treatments; medical sophistication; disruptive hospitalisation; symptoms that shift from one organ system to another; tolerance of painful and invasive procedures without complaint; demands for analgesic medication; and either the absence, or collusion, of visitors" [23]. There is evidence of at least six of these features in our case. Folks and Freeman [24] take a more simplified approach and describe the three 'essential features' of Munchausen's syndrome as 1) recurrent, feigned, or simulated illness; 2) peregrination (travelling or wandering) and; 3) pseudologia fantastica, or pathological lying. All these were present in the case here reported.

Considering the first two essential features, evidence of a recurrent similar presentation and wandering behaviour, both require collateral information from informants, previous records, treating teams or nearby hospitals, as far as the patient allows. National Health Services or social security numbers can be used to cross-check medical records and see if an alias has presented similarly elsewhere [21]. In the case here described, the recurrent wandering presentation was established, fortuitously, through collateral history from the second consultant and a phone call to the other hospital concerned. In addition, information provided by the person named as the next of kin suggests that there may have been at least a third admission.

The third essential feature of Munchausen's, pathological lying, is suggested by a vague and inconsistent history that does not match findings on clinical examination, and by the use of aliases, false addresses and false next of kin details [20]. All of these features were also evident in our patient. He gave a dramatic history of a violent sexual and physical assault. Recommendations in the literature about examination of patients reporting sexual assault suggest that one might expect, among other things, perianal trauma, tears and trauma elsewhere on the body [3]. None of these features were present in the case here reported, nor in that of Humes and Lobo, who suspected Munchausen's syndrome partly due to the absence of perianal trauma [17]. Indeed, the similarities between the two cases, based on age, clinical history and x-ray findings, suggest that we may in fact be describing the same patient. A further interesting finding, which does not appear to fit with the history of an assault, is the presence of paper in the neck of the bottle. This has not been reported previously in the literature and begs the question as to why and how the paper came to be present. It may be possible that this paper was packed into the bottle fragment, prior to insertion by the patient, as an attempt to afford him some protection from internal trauma.

When considering the implications of this case, a key point of interest is the nature of the motivation behind the behaviour of rectal foreign body insertion. In the literature on rectal foreign bodies the emphasis has been on the types of objects involved and their surgical management, rather than reasons behind the behaviour. The motivation is often assumed to be sexual but is rarely discussed in practice, probably due to the sensitivity of the situation and the potential embarrassment for patient and clinician alike. In the case here reported, the precise motivation for the behaviour was difficult to assess for several reasons; the type of history given by the patient, his agitated and demanding interpersonal style, and his discharge against medical advice. Taking into account these and other features of the presentation it seems unlikely that the foreign body insertion was for sexual pleasure and that the patient reported he had been sexually assaulted simply to cover his embarrassment, as has been noted to occur in some previous cases in the literature [8,22].

The aetiological factors in factitious disorders remain poorly understood. Carney and Brown examined the background histories, clinical features and possible underlying motives in a series of 42 factitious disorder patients referred to a specialist unit. The vast majority were emotionally deprived in childhood or adulthood, around two-thirds had attempted suicide or self-harmed, over half had psychopathic personality traits (aggression, substance misuse, criminal record) and half had previously been in caring professions. Over a third of cases were wanderers and this subgroup had more admissions, more psychopathic traits, more severe factitious symptoms (i.e. degree of trauma) and were less likely to attend for follow-up appointments. Less than 10% of cases admitted to their deceptions when challenged [25].

Specifically regarding motives for their behaviour, a key finding was that three-quarters of cases had 'severe and incompletely acknowledged sexual or marital difficulties for which they were...seeking vicarious compensation' [25]. Carney and Brown adopt the view that early deprivation leads to an inability to separate from parental figures and an unstable self-image, which is then re-enacted in their 'ambivalent and destructive relationships with their bodies, illnesses, doctors, hospitals and treatment in general' [25]. The medical team are conceived of as surrogate parental figures and as a means of escape from stressful current life situations. They suggest that masochism from invasive medical procedures is the price to pay in order to maintain a close relationship with a "parental figure". Wandering from hospital to hospital has also been described as being akin to the search for these lost figures [25]. In the case here reported, with its specific and unusual mode of presentation, it is possible that the patient is, at some level, wanting or needing help in relation to unresolved sexual abuse or assault earlier in his life.

The management of Munchausen's Syndrome is understandably challenging and Huffman and Stern describe four key principles [21]. The first is performing only those diagnostic tests that are indicated by objective signs, especially if they are invasive tests. Secondly, maintaining a consistent team approach to the treatment plan, to reduce opportunities for the patient to split the team and cause disruption. Thirdly, setting of clear but compassionate boundaries is essential, whilst reaffirming that the team are aware of the patient's distress and are doing their best to help (albeit with a different view from the patient as to what may be beneficial). Fourthly, the team's frustration with the patient should be refocused on the task of discovering which symptoms are genuine and which are factitious, either by direct observation or further tests [21].

An important concern is whether or not to confront the patient about their behaviour. People presenting with Munchausen's Syndrome are difficult to engage and often react defensively when challenged, resulting in self-discharge and subsequent presentation elsewhere. It is suggested, however, that a 'therapeutic confrontation' may be appropriate in cases where there is a reasonable rapport with the patient and clear evidence of deception. The patient can be informed, in a non-judgemental manner, that severe stress can result in unusual behaviours. More often it will be necessary to offer the patient a legitimate 'face-saving' alternative if they do not admit to their deception. The emphasis should be on offering both medical and psychiatric outpatient follow-up for ongoing support [21].

## Conclusion

The diagnosis suggested for our patient, i.e. Munchausen's Syndrome presenting as rectal foreign body insertion, has only been considered in one other published case and may represent a new variant of the syndrome if further similar cases are reported [26]. In light of this, clinicians should consider the specific motivation for the behaviour in all cases of rectal foreign body insertion, including the possibility of factitious disorder, and avoid any assumption that it has been carried out for sexual gratification. Early involvement of psychiatrists is recommended. Other cases of Munchausen's Syndrome may present which could be identified and addressed more effectively if this approach were taken.

## Competing interests

The authors declare that they have no competing interests.

## Authors' contributions

SK and SC wrote the initial case report and commented on drafts of the paper. CD carried out the literature search on rectal foreign bodies, contributed to the introduction and commented on drafts of the paper. SK and PT carried out the detailed literature search on Munchausen's syndrome and primarily wrote the discussion section of the paper, and PT edited drafts to produce the final version.

## Consent

Because the patient, in this case of Munchausen's Syndrome, gave a false name and address it was not possible to obtain written informed consent from him. We believe this case report contains a worthwhile clinical lesson or public health point which could not be as effectively made in any other way.
